# Supplementary material for: Prognostic Impact of Mucin Expression in Curatively Resected Ampulla of Vater Cancer
Source: Cancers (Basel). 2024 Jun 1;16(11):2120. doi: 10.3390/cancers16112120 (PMC11171239; doi:10.3390/cancers16112120)
Supplement: Supplementary file 1 [file cancers-16-02120-s001.zip › cancers-3026287-supplementary.pdf]

Supplementary Table S1. Expression of mucins in 68 patients with AoV cancer

		Mucin expression (n)							
		MUC1		MUC2		MUC5AC		MUC6	
		-	+	-	+	-	+	-	+
MUC1	-	1	0	1	0	1	0	1	0
	+	0	67	39	28	35	32	37	30
MUC2	-			40	0	20	20	20	20
	+			0	28	16	12	18	10
MUC5AC	-					36	0	28	8
	+					0	32	10	22
MUC6	-							38	0
	+							0	30

-, negative expression; +, positive expression

Supplementary Table S2. Correlation between the expression of mucins and clinicopathologic factors for 68 AoV cancer

	Total	MUC2		MUC5AC		MUC6	
	(n)	+	P-value	+	P-value	+	P-value
Age			0.260		0.254		0.439
≥65	43	15		9		9	
< 65	25	13		23		21	
Sex			1.0		0.841		0.237
Female	21	9		9		12	
Male	47	19		23		18	
T stage			0.437		0.573		0.727
1/2	29	14		12		14	
3/4	39	14		20		16	
N stage			0.896		0.017*		0.371
0	37	16		12		14	
1/2	31	12		20		16	
Differentiation			1.0		0.779		1.0
Others	51	21		23		22	
Poorly differentiated	17	7		9		8	
Lymphovascular Invasion			0.358		0.244		0.896
Negative	38	18		15		16	
Positive	30	10		17		14	
Perineural invasion			1.0		1.0		0.20
Negative	43	18		20		22	
Positive	25	10		12		8	
